# The Effects of Partial Substitution of Fertilizer Using Different Organic Materials on Soil Nutrient Condition, Aggregate Stability and Enzyme Activity in a Tea Plantation

**DOI:** 10.3390/plants12223791

**Published:** 2023-11-07

**Authors:** Chengyi Huang, Kairui Zhang, Wentao Guo, Huijuan Huang, Zhangyong Gou, Liu Yang, Yian Chen, Kokyo Oh, Conggang Fang, Ling Luo

**Affiliations:** 1 College of Water Conservancy and Hydropower Engineering, Sichuan Agricultural University, Ya’an 625014, China; 2 College of Environmental Sciences, Sichuan Agricultural University, Chengdu 611130, China; 3 Saitama International Center for Environmental Science, Saitama 330-9301, Japan; 4 Chengdu Land and Resources Information Center, Chengdu 611130, China

**Keywords:** organic materials, replacement of chemical fertilizer, soil nutrients, soil aggregate enzyme activity, tea plantation

## Abstract

Fertilization plays a crucial role in enhancing tea production. However, it has been demonstrated that the long-term single application of chemical fertilizer will reduce soil nutrient content and the formation of soil aggregates, which is not conducive to the sustainable development of soil and agriculture. Many studies have shown that partial substitution of chemical fertilizer with organic fertilizer can improve soil physicochemical properties and soil nutrient content. This study compared the effects of different organic materials as substitutes for chemical fertilizer. We partially replaced chemical fertilizer with rabbit manure, wine lees and rapeseed cake, amounting to 30% of the total annual nitrogen application in the field experiment, and we set nine different fertilization methods to assess and analyze the soil nutrient condition, aggregate stability and enzyme activity. The results showed that the experimental soil aggregate mean weight diameter (MWD) and geometric mean diameter (GMD) were significantly increased compared with control (*p* < 0.05); the aforementioned fertilization methods also decreased the soil aggregate fractal dimension (D), disruption rate (PAD), average weight-specific surface area (MWSSA) and soil erodibility factor (K). The application of the fertilizer containing organic materials and microbial agent increased soil organic carbon (SOC) by 20.7% to 22.6% and total nitrogen (TN) by 34.6% to 38.1%; it also significantly promoted sucrase, urease and protease activities in all aggregate sizes (*p* < 0.05) and increased the 2–5 mm aggregate content. The correlation coefficients between the SOC and the enzyme activities were 0.18–0.95, and most of them showed an extremely significant positive correlation (*p* < 0.01). In conclusion, the application of fertilizers containing organic materials and microbial agents can improve soil aggregate stability, aggregate enzyme activity and soil structural stability.

## 1. Introduction

Soil aggregates are the basic units of soil structure and important indicators of soil health [1]. Soil function depends on soil structure to a large extent, and the distribution of soil aggregates is the physical component of soil structure [2]. Soil aggregate stability is limited by many factors, such as environmental conditions and fertilizer types [3,4,5]. The application of fertilizers in agricultural production increases soil organic matter content and biological activity, but excessive use of fertilizer increases nitrogen greenhouse gas emissions, exacerbates global warming and is prone to causing groundwater pollution, so it may have negative effects on soil and human health [6,7]. The application of organic fertilizer has significantly increased water holding capacity, porosity, infiltration capacity, hydraulic conductivity and water stable aggregation, at the same time improving soil structure and facilitating the development of agricultural cultivation [8].

Tea is a crucial commercial crop in China, with the tea planting area spanning 18 provinces covering three million hectares in 2019 and increasing by 4.60% each year [9]. The growth of tea plants cannot be separated from the role of soil nutrients and soil aggregates, as tea plants deplete substantial nutrients after multiple harvests, leading to a widespread dependence on excessive chemical fertilizer in pursuit of higher yields [10]. While chemical fertilizers can boost soil organic carbon (SOC) levels, the abuse of chemical fertilizers undermines sustainable land development. In Japanese tea plantations, excessive application of fertilizer has led to soil compaction and agricultural non-point source pollution, resulting in the degradation of soil fertility and structure [11]. Therefore, it is vital to prioritize research aimed at reducing the application of chemical fertilizers [12,13].

SOC and TN are intricately linked to soil aggregates, influenced by a combination of natural and human-induced factors. Different soil aggregates possess varying protective capacities for soil carbon and nitrogen; among these factors, fertilization has the most significant anthropogenic influence on soil [14]. A study by Salehi et al. in Iran shows that both organic and chemical fertilizers can effectively increase SOC and total nitrogen (TN) content, but partial substitution of organic fertilizer can maintain SOC pools and enhance soil nitrogen (N), phosphorus (P) and potassium (K) supply capacity [15]. In particular, it has become evident that SOC exhibits notable increases with the addition of organic fertilizers [16]. On the other hand, the excessive use of chemical fertilizers leads to soil acidification, soil farming difficulties and environmental pollution [17].

The level of soil enzyme serves as a crucial index for testing soil fertility. Soil enzymes play a vital role in catalyzing the conversion of soil nitrogen, carbon and phosphorus, at the same time also influencing the composition of soil microbial communities [18]. According to a study by Dorodnikov et al. in Germany on the influence of soil structure on enzyme activity, the relationships between aggregate and enzyme activity are complex, there are both positive and negative relationships [19]. Fertilization enhances the enzymatic transition of organic compounds, resulting in significant increases in SOC and TN content [20]. However, excessive fertilization causes soil compaction, seriously affects soil respiration and inhibits soil nutrient cycling and organic matter decomposition [21]. Therefore, it is imperative to investigate soil enzyme activity and optimize soil nutrient utilization efficiency.

Sichuan is one of China’s major tea-producing regions, and the land in region uses conventional fertilizers to ensure long-term high yields. However, many studies have shown that excessive application of fertilizer causes soil nutrient imbalance and a series of environmental problems [22,23]. Therefore, the purpose of this study is to explore the effect of different organic materials (rabbit manure, wine lees and rapeseed cake) replacing chemical fertilizers on the improvement of soil nutrients, soil structure and enzyme activity. The purpose of this study is twofold: firstly, to explore the short-term effects of organic materials combine with fertilizer on soil nutrients and soil structure; secondly, to explore the effects of different organic materials and microbial agents combine with fertilizer on soil structure and nutrients. Correlation analysis and principal component analysis are carried out to determine the effects of different organic materials and select the best organic materials, providing suggestions and references for the formulation of efficient and sustainable fertilization measures.

## 2. Materials and Methods

### 2.1. Experimental Site and Treatments

The experimental site was situated in Mingshan District, within Ya’an City, Sichuan Province, on the western edge of the Sichuan Basin. It experiences an average annual temperature of 15.4 °C, approximately 1500 mm of annual rainfall, a frost-free period lasting 298 days, 1018 h of annual sunshine and maintains an average annual relative humidity of 82%. The natural vegetation in this area comprises mid-subtropical evergreen broad-leaved forest, with a forest cover of roughly 32% [24], The soil basic physical and chemical properties are shown in Table 1. According to the third edition of the International Soil Classification System (WRB) proposed by Peter Schad in 2014 [25], the soil in the experimental area is classified as yellow soil, originating from ancient alluvial deposits of the fourth geological system, The basic characteristics of 0–15 cm and 15–30 cm soil layers are shown in Table 1.

This experiment covered a tea growing period from October 2019 to October 2020. We used three organic materials (rabbit manure, wine lees and rapeseed cake) to partially substitute chemical fertilizer, amounting to 30% of the total annual nitrogen application. This resulted in nine distinct fertilization modes: CK (no fertilizer), CF (full chemical fertilizer alone), CFM (full chemical fertilizer combined with microbial agent), CFD (70% chemical fertilizer combined with rabbit manure), CFR (70% chemical fertilizer combined with rapeseed cake), CFL (70% chemical fertilizer combined with wine lees), CFDM (70% chemical fertilizer combined with rabbit manure and microbial agent), CFRM (70% chemical fertilizer combined with rapeseed cake and microbial agent) and CFLM (70% chemical fertilizer combined with wine lees and microbial agent). All treatments were matched for N, P and K content (Table 2). Each treatment was replicated three times, resulting in a total of 27 treatment zones, each covering 20 m^2^. These zones were randomly distributed [26]. Field management modes were consistent except for CK; urea, calcium superphosphate and agricultural potassium sulfate served as the test fertilizers, with urea containing 46% nitrogen, calcium superphosphate containing 12% phosphorus pentoxide (P_2_O_5_) and agricultural potassium sulfate containing 50% potassium oxide (K_2_O) [27].

The tea species cultivated in the experimental tea plantation was Fuyun No. 4, with a plant spacing of 30 cm and rows spaced approximately 150 cm apart. Fertilization standards were annual application of nitrogen fertilizer 400 kg hm^−2^, phosphorus fertilizer 150 kg hm^−2^ and potash 200 kg hm^−2^ [28]. Fertilization application measures were as follows: the CK treatment did not apply any fertilizer, 70% of the total nitrogen fertilizer was applied to as the basal fertilizer, the remaining 30% of the nitrogen fertilizer was applied as the additional fertilizer at a ratio of 2:1:1 in the spring and summer and autumn seasons, and all the phosphorus and potash fertilizers were applied in the basal fertilizer at the end of October 2019 along the drip line of the tea rows to open the depth of about 15cm fertilizer furrow, and combined with irrigation in a uniform mulching application [29]. Spring, summer and autumn fertilizer in 2020 was applied, respectively, in early February, mid-May and early July, for the follow-up application. In the treatments of organic materials with chemical fertilizers, organic materials with 30% of the total annual nitrogen content were applied to replace the chemical fertilizer in the one-time application of the base fertilizer, the remaining 40% of the total annual nitrogen was applied to make up for the urea, and the last remaining 30% of nitrogen fertilizer was replaced with organic materials in the ratio of 2:1:1 in the spring, summer and autumn as a follow-up fertilizer application. The microbial agent effective number of live bacteria was greater than 2 × 10^10^ g^−1^; the main strains of microbial agents used were *Bacillus licheniformis*, *Bacillus subtilis*, *Bacillus amyloliquefaciens*, actinomycetes, green wood mould, etc., diluted in a concentration of 1:800. In the fertilization using clear liquid spraying and sludge watering roots, the application amount of 7.5 kg hm^−2^ and microbial agent did not include the content of nitrogen, phosphorus and potassium [30]. The CF and CFM treatments applied fertilizer throughout the year, and the total amounts of nitrogen, phosphorus and potassium applied to the base fertilizer was consistent with the respective organic material dosing treatments; the pattern of follow-up fertilizer use was consistent with the respective dosing treatments of organic materials.

### 2.2. Soil Sample Collection and Aggregate Fraction

Soil samples were collected at the end of October 2020, and the soil was collected from 0–15 cm and 15–30 cm soil layers at 10–20 cm from the fertilization furrow of the tea tree using the multi-point mixed sampling method. The soil samples were then removed using the quadrat method after mixing, and the samples were transported back to the laboratory in a refrigerated plastic bag. The plant and animal debris as well as gravel of the soil samples were picked out, and some of the samples were sieved through a 2 mm sieve and placed in a refrigerator at 4 °C for spare parts, while the rest of the soil samples were air-dried and prepared for use [31].

After the soil samples were completely air-dried, a portion of the fresh samples was taken and stored at 4 °C for the assay of soil enzyme activities; the other portion, which had been weighed, dried at 105 °C and weighed for the assay of SOC and TN content, was collected into aluminum boxes. The calculation of the soil aggregate index was performed according to Elliott’s (1986) [32] wet sieving method and Six’s (1998) [33] dry sieving method for soil aggregates, which were 5 mm, 2 mm, 1 mm, 0.5 mm and 0.25 mm, according to the size of the aggregate from the largest to the smallest [34]. Sufficient soil aggregate samples were collected, and the parameters of their structural characteristics, such as soil aggregate mean weight diameter (MWD), geometric mean diameter (GMD), average weight-specific surface area (MWSSA), disruption rate (PAD), erodibility factor (K) and fractal dimension (D) values were calculated [35,36,37].

The specific formula are:

$$MWD=\sum \left(M_{i}/M_{T}\right){\bar{X}}_{i}$$

$$GMD=\exp\left\{\sum \left(M_{i}/M_{T}\right)\ln\bar{X_{i}}\right\}$$

$$\mathrm{MWSSA}=\overset{n}{\underset{i=1}{\sum }}\frac{6M_{i}}{2.65{\bar{X}}_{i}}$$

$$PAD=\left(DR_{0.25}-WR_{0.25}\right)/DR_{0.25}\times 100\%$$

$$K=7.954\times \left\{0.0017+0.0494\times \exp\left[-0.5\times {\left(\frac{1.675+\mathrm{lg}GMD}{0.6986}\right)}^{2}\right]\right\}$$

where ${\bar{X}}_{i}$ denotes the mean diameter (mm) of the aggregates in any aggregate size (i) fraction, $M_{i}$ denotes the weight of a size class (i) to the total weight, $M_{T}$ denotes the aggregate total weight, $DR_{0.25}$ denotes the aggregate weight greater than 0.25 mm by dry sieving method, and $WR_{0.25}$ denotes the aggregate weight greater than 0.25 mm by wet sieving method.

D is calculated using the formula:

$$\frac{M\left(\delta <\bar{X_{i}}\right)}{M_{T}}={\left(\frac{\bar{X_{i}}}{{\bar{X}}_{max}}\right)}^{3-D}$$

According to the formula, taking $\ln\left\{M\left(\delta <\bar{X_{i}}\right)/M_{T}\right\}$ and $\ln\left(X_{i}/{\bar{X}}_{max}\right)$ as the vertical and horizontal coordinates, the slope (3 − D) is obtained, and D value is obtained, where $M_{i}\left(\delta <\bar{X_{i}}\right)$ denotes the cumulative mass of aggregate size less than ${\bar{X}}_{i}$, and ${\bar{X}}_{max}$ denotes the average diameter of the largest aggregate size.

### 2.3. Measurement of Soil Nutrients

Soil organic carbon was estimated using the dilution heat method with potassium dichromate and sulfuric acid [24]; soil total nitrogen was evaluated via the macro Kjeldahl digestion procedure [38].

Soil aggregate enzyme activities (sucrase, protease, urease) were determined using the colorimetric method with 3,5-dinitrosalicylic acid for soil sucrase activity in soil aggregates [39], soil aggregate protease activity was determined using the colorimetric method with ninhydrin [40] and soil aggregate urease activity was determined with the colorimetric method using sodium phenol–sodium hypochlorite [20].

### 2.4. Statistical Analysis

All statistical calculations were carried out using SPSS 22.0 for initial data analysis and one-way analysis of variance (ANOVA) and Origin 2022 for graphing. Significant differences between treatments were tested using multiple comparisons and the least significant difference method, with different lowercase letters indicating significant differences between treatments (*p* < 0.05). Pearson’s correlation was used to evaluate the relationship between soil aggregate stability indexes and soil nutrient content. At the same time, the soil aggregate size distribution, soil nutrient content and soil aggregate enzyme activity of soil aggregates were analyzed via principal component analysis.

## 3. Results

### 3.1. Soil Water Stability Aggregate Distribution

**Figure 1:**
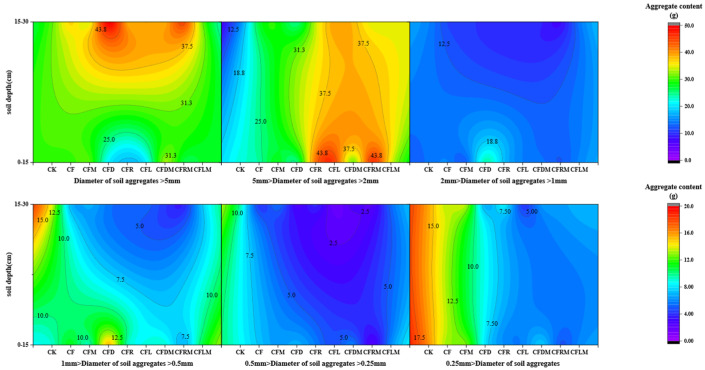
Distribution of water-stable aggregates with different sizes in the 0–15 cm depth and 15–30 cm depth of soil. Note: CK: no fertilizer; CF: full chemical fertilizer alone; CFM: full chemical fertilizer combined with microbial agent; CFD: 70% chemical fertilizer combined with rabbit manure; CFR: 70% chemical fertilizer combined with rapeseed cake, CFL: 70% chemical fertilizer combined with wine lees; CFDM: 70% chemical fertilizer combined with rabbit manure and microbial agent; CFRM: 70% chemical fertilizer combined with rapeseed cake and microbial agent; and CFLM: 70% chemical fertilizer combined with wine lees and microbial agent (the same below).

In the range of >0.25 mm aggregate size, the soil aggregate content of all treatments showed a decreasing trend with the decrease in aggregate size, but the soil aggregate content of <0.25 mm aggregate size suddenly increased (Figure 1). The highest soil aggregate content was observed in the >5 mm and 2–5 mm fractions, ranging from 18.26% to 49.52% and from 15.02% to 46.05%. Specifically, in the first soil layer (0–15 cm), CFDM, CFRM and CFLM significantly increased the 2–5 mm aggregate content and reduced the content of <0.25 mm and 0.25–0.5 mm aggregates compared to CK. In the second soil layer (15–30 cm), all treatments enhanced the 2–5 mm and >5 mm aggregate content while decreasing the other aggregate content. Each treatment played a role in adjusting the distribution of different aggregate sizes; the primary effect was the reduction in 0.25–0.5 mm and <0.25 mm aggregates and increase in other aggregates, ultimately leading to an improvement in soil structure.

### 3.2. Physical Indexes of Soil Aggregate

**Figure 2:**
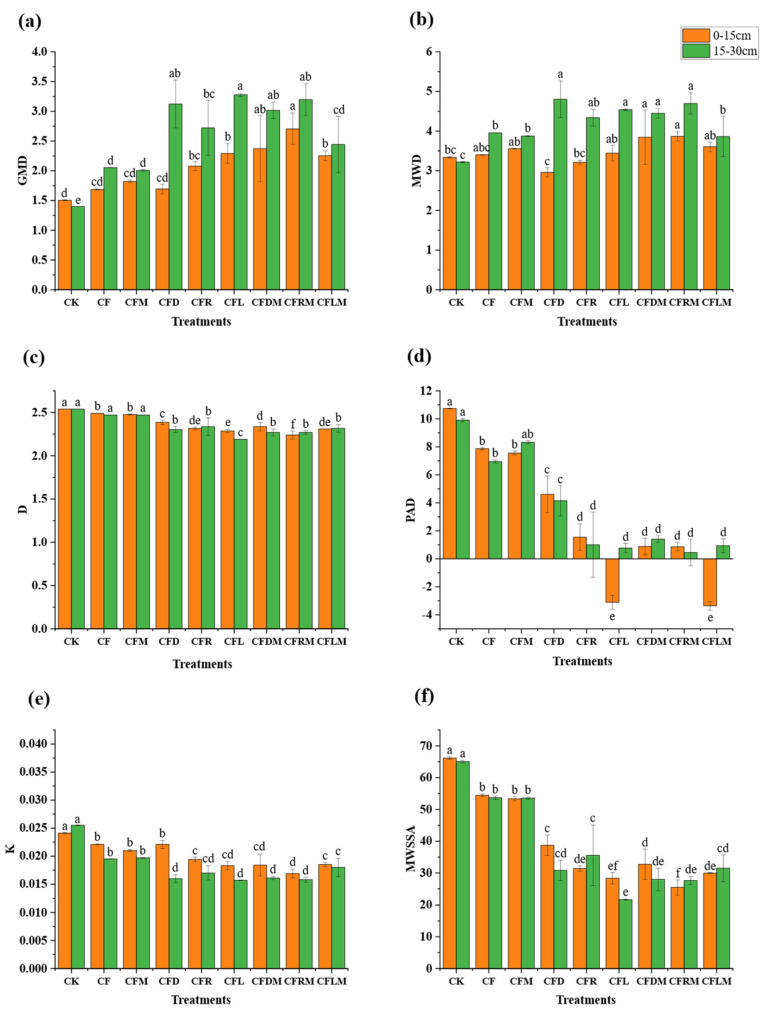
The value of GMD (**a**), MWD (**b**), D (**c**), PAD (**d**), K (**e**) and MWSSA (**f**) under different treatments in the 0–15 cm depth and 15–30 cm depth of soil. Note: MWD: weight diameter of aggregates; GMD: geometric mean diameter of aggregates; D: fractal dimension of aggregates; MWSSA: average weight-specific surface area of aggregates; PAD: aggregate disruption rate; K: soil corrosivity factor of aggregates. Different column colors mean different soil depths, the bars stand for mean ± SD, and different small letters in the column mean significant difference in aggregate indexes at 0.05 level among treatments (the same below).

Higher values of GMD and MWD indicate better soil stability and soil aggregation. The greatest impacts on GMD and MWD values were observed in the CFRM and CFDM treatments. In the first soil layer, GMD increased by 52.4% to 79.8%, while MWD increased by 15.2% to 15.8% compared to CK. In the second soil layer, GMD increased by 115.7% to 128.6% and MWD saw an increase of 38.2% to 49.2% (Figure 2a,b). Most treatments involving the combination of organic materials induced a significant increase in GMD and MWD, with the effect being more remarkable in treatments including microbial agents. In the first soil layer, other treatments showed significantly different GMD from CK, while MWD was only significantly different for CFRM and CFDM compared to CK (*p* < 0.05). In the second soil layer, both GMD and MWD for all treatments were significantly different from CK (*p* < 0.05).

Larger values of D and PAD indicate poorer soil stability, while higher values of K and MWSSA suggest a greater potential for soil erosion. The most substantial impact on D, PAD, K and MWSSA values was observed in the treatments involving organic materials and microbial agents. In the first soil layer, the treatments induced a reduction in D by 8% to 11.8%, PAD by 91.8% to 131.2%, K by 23.3% to 29.9% and MWSSA by 50.6% to 61.6% compared to the CK treatment. In the second soil layer, D values decreased by 8.8% to 13.8%, PAD decreased by 85.8% to 95.5%, K decreased by 29.5% to 38.5% and MWSSA decreased by 51.6% to 66.6%; all treatments which added organic materials reduced the values of D, PAD, K and MWSSA (Figure 2c–f). Fertilization had a more substantial impact on the PAD and D values, and the treatments that replaced the chemical fertilizer with organic materials were significantly different from CK (*p* < 0.05).

### 3.3. Soil Organic Carbon and Total Nitrogen Content

**Figure 3:**
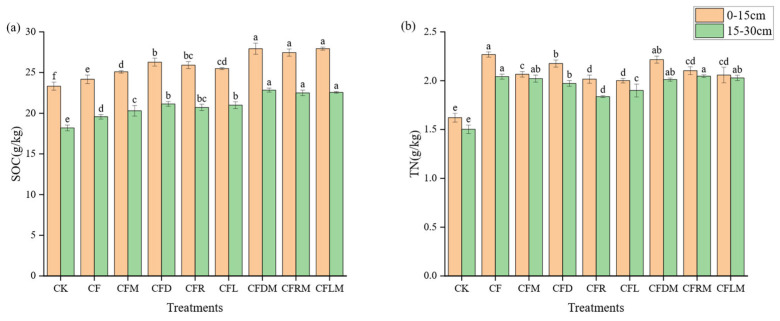
SOC content (**a**) and TN content (**b**) under different treatments in the 0–15 cm depth and 15–30 cm depth of soil. Note: SOC: soil organic carbon (g/kg); TN: total nitrogen (g/kg). Different column colors mean different soil depths, the bars stand for mean ± SD, and different small letters in the column mean significant difference in aggregate indexes at 0.05 level among treatments, (the same below).

Notably, SOC and TN content was higher in the first soil layer compared to the second soil layer, with elevations ranging from 21.4% to 28.1%. As for SOC content, in the first soil layer, CFLM, CFDM and CFRM treatments led to an increase of 17.70% to 19.78% compared to CK; in the second soil layer, they resulted in an increase of 23.7% to 25.5% (Figure 3a). Regarding TN content, the CF and CFD treatments showed the higher values in the first soil layer, surpassing CK by 34.2% to reach 39.9%; in the second soil layer, CF and CFRM treatments had an increase of 35.0% to 36.2%. Most treatments resulted an increase of nearly 30% in TN content (Figure 3b). It is worth noting that in the same treatment, TN content varied slightly in different soil layers, exhibiting an increase of 1.47% to 11.09% with the increase in soil depth.

In terms of SOC content, organic materials that replaced chemical fertilizer had a stronger effect, with microbial agents playing a reinforcing role. On the other hand, for TN content, the CF treatment was significantly higher than the other treatments.

### 3.4. Soil Aggregate Enzyme Activity

**Figure 4:**
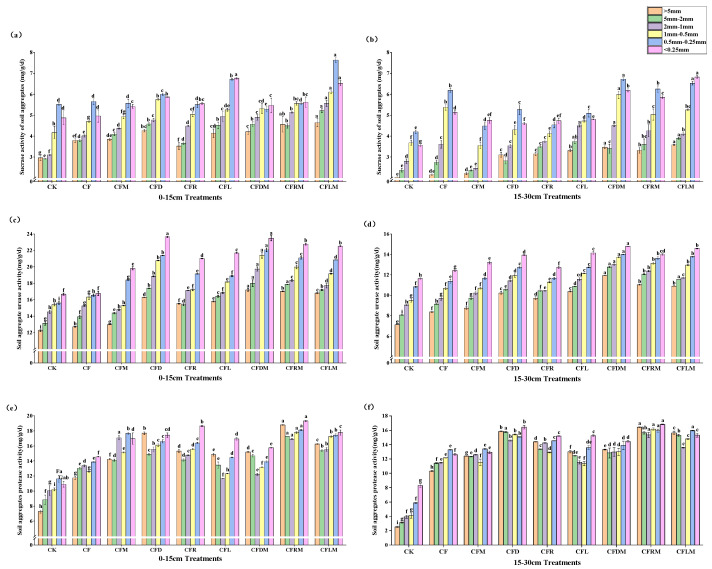
Soil aggregate sucrase activity values in the 0–15 cm depth (**a**) and 15–30 cm depth (**b**) of soil, urease activity values in the 0–15 cm depth (**c**) and 15–30 cm depth (**d**) of soil, protease activity values in the 0–15 cm depth (**e**) and 15–30 cm depth (**f**) of soil. Different small letters in the column mean significant difference in aggregate indexes at 0.05 level among treatments, different column colors mean different aggregate sizes, and the bars stand for mean ± SD.

Sucrase plays a crucial role in increasing the solubility of soil nutrients, urease is closely linked to soil nitrogen supply, and soil protease serves as the rate-limiting enzyme in the soil nitrogen mineralization process. The sucrase and urease activities gradually increased as aggregate size decreased, peaking at the <0.25 mm and 0.25–0.5 mm aggregate fractions (Figure 4a–d). In contrast, there was no clear regularity in the variation in protease activities with different aggregate sizes (Figure 4e,f). The replacement of chemical fertilizer with organic materials significantly enhanced sucrase, urease and protease activity. Overall, the performance ranking of different treatments was as follows: organic materials + microbial agent + chemical fertilizer > organic materials + chemical fertilizer > chemical fertilizer + microbial agent > CF > CK. Among sucrase and urease, the CFDM, CFRM and CFLM treatments had the most remarkable effect in enhancing their activities in soil aggregates (*p* < 0.05). As for protease, the CFD, CFRM and CFLM treatments exhibited the most significant promotion in soil aggregates (*p* < 0.05). Across all soil layers, all aggregate sucrase, urease and protease activities were generally significantly higher as the soil depth increased. There were significant differences in urease activities between the two soil layers.

### 3.5. Correlation Analysis of Indexes of Aggregates

**Figure 5:**
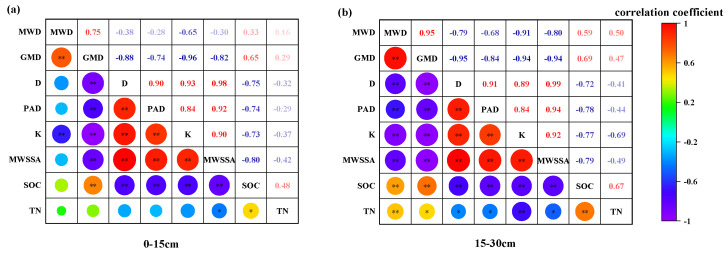
Correlation analysis of MWD, GMD, D, MWSSA, PAD, K, SOC and TN in the 0–15 cm depth (**a**) and 15–30 cm depth (**b**) of soil. Note: “*” indicates significant differences according to the Pearson test (*p* < 0.05), and “**” indicates highly significant differences according to the Pearson test (*p* < 0.05).

There was a highly significant positive correlation (*p* < 0.01) among soil aggregate indexes with correlation coefficients of 0.84–0.99, whereas SOC showed a highly significant negative correlation (*p* < 0.01) with other aggregate indexes, with correlation coefficients ranging from −0.96 to −0.72. MWD was negatively correlated with other indexes in the first soil layer and showed correlation coefficients from −0.65 to −0.28 (Figure 5a), whereas they were highly significantly negatively correlated (*p* < 0.01) in the second layer with correlation coefficients from −0.91 to −0.68 (Figure 5b). TN and SOC were highly significantly correlated (*p* < 0.01) with GMD and MWD, while TN was highly significantly positively correlated with SOC (*p* < 0.01).

It was observed that the correlation coefficients between SOC and the activity of the three enzymes were higher than TN. In the first soil layer, the correlation coefficients between SOC, TN and sucrase for aggregates of <0.5 mm suddenly shifted to negative values. In the second soil layer, the correlation coefficients between TN and the activity of the three enzymes for <0.5 mm aggregates all experienced a notable decrease, while the correlation coefficients for SOC remained unchanged (Table 3).

Analyzing the correlation coefficients between the activity of the three enzymes and SOC and TN, it became apparent that the correlation coefficients between SOC and the activities of the three enzymes exhibited a highly significant positive correlation (*p* < 0.01). In contrast, the correlation coefficients between TN and the activities of the three enzymes showed either no significant correlation or a significant correlation (*p* < 0.05). This suggested that SOC plays a pivotal role in affecting soil enzyme activities, while the impact of SOC on soil enzyme activities is stronger than that of TN.

### 3.6. Principal Component Analysis of Soil Carbon and Nitrogen and Enzyme Activity

**Figure 6:**
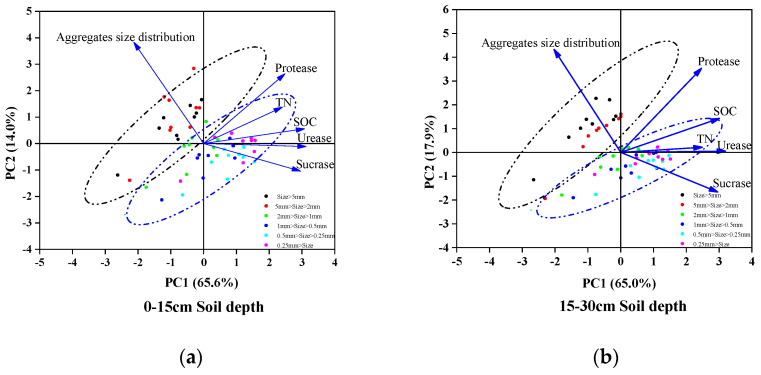
Principal component analysis of different soil aggregate sucrase, urease and protease with TN, SOC and soil aggregate distribution for different treatments in the 0–15 cm depth (**a**) and 15–30 cm depth (**b**) of soil.

In the first soil layer, the first principal component (PC1) and second principal component (PC2) explained 65.6% and 14.0% of the total variation (Figure 6a), respectively, while PC1 and PC2 accounted for 65.0% and 17.9% of the total variation in the second soil layer (Figure 6b). The figure illustrates that the similarity between 2–5 mm and >5 mm aggregates was relatively high, while other aggregates exhibited stronger similarities. Significant differences in PC1 and PC2 were observed for >2 mm and <2 mm aggregates, which suggested that aggregate size has a notable influence on enzyme activities, SOC, TN and aggregate distribution. Sucrase displayed a positive correlation with PC1 and a negative correlation with PC2. Additionally, the different aggregate sizes exhibited a negative correlation with PC1 and a positive correlation with PC2. Consequently, sucrase, urease and SOC played a major role in determining PC1, whereas aggregate distribution was the primary factor influencing PC2.

## 4. Discussion

### 4.1. Effect of Application with Different Organic Materials on the Stability of Soil Aggregates

In this study, most treatments led to an increase in the 2–5 mm aggregate fraction while reducing the proportion of the 0.25–0.5 mm and <0.25 mm aggregates. These findings align with the results from Wang et al. [41], indicating that these three aggregate fractions are more stable. Notably, the CFRM treatment consistently demonstrated the most favorable effect at all aggregate sizes in treatments. This result could be attributed to differences in nutrient content between rapeseed cake and other materials, as well as the catalytic effect of the microbial agent [42]. The effect of each treatment on aggregates was notably stronger than the shallower layer. However, in the first layer, the MWD and GMD values of CFD and CFL treatments were lower than CK. This can be attributed to factors such as soil tillage and rainfall-induced infiltration [43]. Previous studies have indicated that topsoil is more vulnerable to tillage abrasion and the impact of precipitation [44]. Regarding D, PAD, K and MWSSA values, the effect of CFRM and CFL treatments was more significant, and the effect of the microbial agent added to treatments was stronger. The PAD values of CFL and CFLM treatments in the first soil layer even became negative; this phenomenon may be due to the unique effects of wine lees and the enhancement effect of microbial agents [45,46]. The effect of adding the microbial agent was stronger than not adding it, but the effect of CFLM was slightly inferior to that of CFL, which may be due to the fermentation reaction between a small part of distiller’s grains and microbial agent, thus affecting the experimental results, which is consistent with the study of Lv Haofeng et al. [45]. In the principal component analysis, >2 mm aggregates were significantly distinguished from other size aggregates, indicating that small-size aggregates were more conducive to microbial richness and diversity and could help to improve soil enzyme activity in small size aggregates [47]. It is worth noting that organic fertilizer combined with chemical fertilizer effectively improved soil structure, and the microbial agent reshaped the soil bacterial and fungal ecology, as highlighted in the research of Li et al. [48].

Overall, the substitution of organic materials had a more positive impact than using chemical fertilizer alone; the inclusion of microbial agent significantly enhanced soil fertility and improved soil structure.

### 4.2. Effects of Application of Different Organic Materials on Soil Organic Carbon and Total Nitrogen

In this study, the SOC content in treatments involving organic materials was higher than CF. It was challenging to maintain SOC content in the CF application, whereas the addition of organic materials significantly improved soil nutrients [49]. Importantly, the treatments with microbial agents had a more remarkable effect; this may be attributed to the ability of microbial agents to boost soil biological activities and accelerate the conversion of organic materials into SOC. Research by Bei et al. [50] has also demonstrated that organic fertilizer can enhance the capacity of retaining fertility. The treatments in this study substantially increased TN content, with CF being the most effective method for enhancing TN content [51]. This indicates that the CF application is more effective than the use of organic materials for soil nitrogen availability. However, nitrogen from chemical fertilizer can be susceptible to runoff due to rainfall and biological activities [52], whereas nitrogen released from organic materials has a slower release mode, which can maintain soil nitrogen content more effectively. This observation aligns with the research results of Zhai et al. [53], as the TN content of treatments involving organic materials was lower than the CF application and most remarkable in the CFD and CFDM treatments. This suggests that rabbit manure had a significant impact on soil nitrogen content. This difference might be attributed to the unique microbial community composition of manure compost, which is known for its higher diversity compared to other fertilizers, as indicated by Gomes et al. [54]. Through correlation analysis and principal component analysis, we showed that the correlation coefficient between SOC and other indexes is greater than that of TN, which may be due to the close relationship between SOC and soil organic matter, so that it can promote the consolidation of small-size soil aggregates to form large aggregates, thus affecting the soil structure index. This is consistent with the results of Ranjan Bhattacharyya et al. [55]. The correlation coefficient and significance of each index in the first soil layer with SOC and TN were stronger than those in the second soil layer, which may be due to the fact that the surface soil is vulnerable to external disturbance and rainfall erosion. With the increase in soil depth, the stability of soil aggregates increases correspondingly, resulting in differences between different soil layers [56].

Overall, the combination of organic materials with fertilizer had a more significant effect on enhancing SOC content, especially when microbial agents were added. However, for TN content, the CF application had the most pronounced impact on enhancement.

### 4.3. Effect of Different Organic Materials on Soil Sucrase, Urease and Protease Enzyme

In this study, sucrase, urease and protease displayed the higher enzyme activities in microaggregates (<0.25 mm). Specifically, sucrase and urease enzyme activities exhibited a gradual increase with decreasing aggregate sizes; this phenomenon might be attributed to the enzyme activities relied on the distribution of SOC [57]. Macroaggregates (>0.25 mm) tended to contain higher SOC content, which resulted in higher sucrase and urease activities in microaggregates. Protease exhibited complex changes across different aggregates due to variations in the SOC distribution [58]. This suggests that macroaggregates had a lesser impact on protease, a finding consistent with previous research by Jiang et al. [59], who found that microbial numbers and enzyme activities were higher in microaggregates compared to macroaggregates [47]. The addition of microbial agents strengthened microbial activities and accelerated the decomposition of soil organic materials, contributing to the enhancement of soil enzyme activities [60]. Notably, enzyme activities in the first soil layer were higher than the second layer; this discrepancy could be attributed to the favorable hydrothermal conditions and aeration present in surface soil, which created a more conducive environment for enzyme activity [61]. This aligns with the findings of Xu et al. [62], who noted that microbial agents promote the favorable environmental conditions and provide energy and nutrients for microbial growth. Among the organic materials treatments without microbial agent, the rabbit manure treatment exhibited the highest protease activity. However, the highest protease activity was found in the rapeseed cake treatment with the microbial agent. These differences may be attributed to varying reactions between the three organic materials and microbial agent [63]. The principal component analysis showed that protease, sucrase and urease were positively correlated with the first principal component in different soil aggregate sizes. The SOC and TN of most aggregates were positively correlated with sucrase, urease and protease activities, and the correlation coefficient between sucrase, urease and protease activities and SOC was higher than that of TN. This may be because different aggregate sizes have different effects on SOC and TN, and the close relationship between SOC and soil organic matter made the correlation between enzyme activity and organic carbon greater, which is consistent with previously published results [64,65].

This suggests that the application of organic materials in combination with fertilizer is more effective in enhancing the enzyme activity of aggregates, and the addition of microbial agents can further enhance soil aggregate enzyme activity.

## 5. Conclusions

Under the conditions of this experiment, all treatments improved soil structure, nutrient status and enzyme activity. The applications which combined organic materials with microbial agents significantly increased the values of MWD, GMD, SOC, TN and three enzyme activities of soil aggregates, and decreased the values of D, PAD, K and MWSA of soil aggregates. The effect of adding microbial agents was stronger than that of not adding microbial agents. Among them, the CFRM treatment has the most significant effects on the aggregate physical indexes, SOC, and activities of sucrase, urease and protease. Although the TN content of the CFRM treatment was slightly lower than that of the CF treatment, the overall effects of the CFRM treatment on soil aggregates, enzyme activity and SOC were significantly stronger than those of the CF treatment. The research results indicated that partial replacement of chemical fertilizer with organic materials was beneficial for the improvement of soil structure and fertility level in the tea garden as a whole, and improved the long-term sustainability of soil fertility, thereby improving soil and ecosystem function.

## Figures and Tables

**Table 1:** Basic physical and chemical properties of soil.

Soil Depth	pH	SOC	TN	AN	AP	AK
0–15 cm	3.97	21.51	1.84	142.27	23.53	240.11
15–30 cm	4.40	18.83	1.63	117.48	18.54	192.29

SOC: soil organic carbon (g·kg^−1^); TN: total nitrogen (g·kg^−1^); AN: Alkali-hydrolyzed nitrogen (mg·kg^−1^); AP: Available phosphorus (mg·kg^−1^); AK: Available Potassium (mg·kg^−1^).

**Table 2:** Nutrients content of applied organic materials.

Organic Material Type	Water Content (%)	Total Nitrogen (%)	Total Phosphorus (%)	Total Potassium (%)
Rabbit manure	56.15	2.25	0.79	1.73
Rapeseed cake	45.12	5.94	0.82	1.13
Wine lees	75.42	3.36	0.59	0.80

**Table 3:** Correlation analysis of SOC, TN, sucrase, urease and protease at different soil aggregate sizes in the 0–15 cm depth and 15–30 cm depth of soil.

Soil Depth	Enzyme Species		Soil Aggregate Sizes (mm)					
			>5	5–2	2–1	1–0.5	0.5–0.25	<0.25
0–15 cm	Sucrase	SOC	0.82 **	0.74 **	0.83 **	0.79 **	0.18	0.36
		TN	0.55 **	0.60 **	0.44 *	0.39 *	−0.19	0.00
	Urease	SOC	0.90 **	0.87 **	0.86 **	0.88 **	0.95 **	0.81 **
		TN	0.33	0.50 **	0.48 *	0.45 *	0.44 *	0.36
	Protease	SOC	0.81 **	0.82 **	0.36	0.62 **	0.54 **	0.67 **
		TN	0.64 **	0.68 **	0.40 *	0.32	0.24	0.41 *
15–30 cm	Sucrase	SOC	0.89 **	0.72 **	0.73 **	0.66 **	0.75 **	0.84 **
		TN	0.31	0.42 *	0.39 *	0.39 *	−0.31	0.00
	Urease	SOC	0.97 **	0.97 **	0.94 **	0.95 **	0.97 **	0.94 **
		TN	0.57 **	0.67 **	0.60 **	0.60 **	−0.16	0.11
	Protease	SOC	0.80 **	0.81 **	0.73 **	0.73 **	0.71 **	0.70 **
		TN	0.68 **	0.86 **	0.84 **	0.91 **	0.38	0.65 **

Note: SOC: soil organic carbon; TN: total nitrogen. “*” indicates significant differences according to the Pearson test (*p* < 0.05), “**” indicates highly significant differences according to the Pearson test (*p* < 0.05).

## Data Availability

Data are available on request due to restrictions, e.g., privacy-based or ethical.
